# Methylene Blue Preserves Cytochrome Oxidase Activity and Prevents Neurodegeneration and Memory Impairment in Rats With Chronic Cerebral Hypoperfusion

**DOI:** 10.3389/fncel.2020.00130

**Published:** 2020-05-20

**Authors:** Allison M. Auchter, Douglas W. Barrett, Marie H. Monfils, F. Gonzalez-Lima

**Affiliations:** Department of Psychology, Institute for Neuroscience, The University of Texas at Austin, Austin, TX, United States

**Keywords:** methylene blue, chronic cerebral hypoperfusion, carotid artery occlusion, cytochrome oxidase, interregional correlations, vascular hypothesis of Alzheimer’s dementia

## Abstract

Chronic cerebral hypoperfusion in neurocognitive disorders diminishes cytochrome oxidase activity leading to neurodegenerative effects and impairment of learning and memory. Methylene blue at low doses stimulates cytochrome oxidase activity and may thus counteract the adverse effects of cerebral hypoperfusion. However, the effects of methylene blue on cytochrome oxidase activity during chronic cerebral hypoperfusion have not been described before. To test this hypothesis, rats underwent bilateral carotid artery occlusion or sham surgery, received daily 4 mg/kg methylene blue or saline injections, and learned a visual water task. Brain mapping of cytochrome oxidase activity was done by quantitative enzyme histochemistry. Permanent carotid occlusion for 1 month resulted in decreased cytochrome oxidase activity in visual cortex, prefrontal cortex, perirhinal cortex, hippocampus and amygdala, and weaker interregional correlation of cytochrome oxidase activity between these regions. Methylene blue preserved cytochrome oxidase activity in regions affected by carotid occlusion and strengthened their interregional correlations of cytochrome oxidase activity, which prevented neurodegenerative effects and facilitated task-specific learning and memory. Brain-behavior correlations revealed positive correlations between performance and brain regions in which cytochrome oxidase activity was preserved by methylene blue. These results are the first to demonstrate that methylene blue prevents neurodegeneration and memory impairment by preserving cytochrome oxidase activity and interregional correlation of cytochrome oxidase activity in brain regions susceptible to chronic hypoperfusion. This demonstration provides further support for the hypothesis that lower cerebral blood flow results in an Alzheimer’s-like syndrome and that stimulating cytochrome oxidase activity with low-dose methylene blue is neuroprotective.

## Introduction

Aging and dementia involve progressive reduction in cerebral blood flow and energy metabolism that result in cognitive dysfunction (de la Torre, 1999; Farkas and Luiten, 2001). Patients with cerebrovascular disorders and late-onset Alzheimer’s disease (AD) show marked cerebral hypoperfusion (de la Torre, 2012). Even normal aging-related reduction in cerebral blood flow results in significant functional pathology when combined with other factors such as cardiovascular disease (Haley et al., 2007) and cerebrovascular ischemia (de la Torre et al., 1997; Cada et al., 2000). Interestingly, in AD patients who have not suffered cerebral infarction, cerebral perfusion is reduced more symmetrically, globally and chronically than in stroke patients (Tachibana et al., 1984), implicating chronic cerebral hypoperfusion in the pathogenesis of AD (de la Torre, 2018).

Permanent bilateral carotid artery occlusion (2-vessel occlusion; 2VO) is a useful model for the reproduction of chronic cerebral hypoperfusion as it occurs in human aging and AD (de la Torre et al., 1992; de la Torre, 1999, 2000; Farkas et al., 2007). Typically, in rats permanent vessel occlusion does not result in reperfusion injury (as a result of instant recovery of perfusion). Additionally, cerebral hypoperfusion is global, damage to nervous tissue is less dramatic, and there are no obvious signs of motor dysfunction or seizures (de la Torre et al., 1992; Farkas et al., 2007). When neurons are starved of glucose and oxygen via chronic cerebral hypoperfusion, the impending result is mitochondrial dysfunction. If mitochondria do not receive enough glucose and oxygen, electrons from the electron transport chain used to drive ATP synthesis are taken up by other molecules, resulting in the formation of damaging reactive oxygen species (Rojas et al., 2012).

We hypothesized that certain properties of low-dose methylene blue (MB) could be neuroprotective under conditions of reduced supply of glucose and oxygen such as in chronic cerebral hypoperfusion (Gonzalez-Lima and Auchter, 2015). Under physiological conditions in nervous tissue, nearly all the electrons donated to the electron transport chain are derived from the transformation of glucose for the generation of the electron donors NADH and FADH (Erecinska and Silver, 1989). However, MB at low doses reaches a redox equilibrium inside mitochondria that allows MB to cycle electrons directly into the electron transport chain (Rojas et al., 2012). In this way, MB could compensate for a reduced supply of glucose to the brain by becoming an alternative source of electrons donated to the electron transport chain. Cytochrome oxidase (CO) is the last enzyme in the electron transport chain that passes the electrons to oxygen as the final electron acceptor (Erecinska and Silver, 1989). However, there is evidence that MB can maintain energy production even under hypoxic conditions (Lee and Urban, 2002) because MB can replace oxygen by accepting electrons from CO at the end of the electron transport chain (Rojas et al., 2012). Therefore, maintaining CO activity by MB’s electron cycling could compensate for 2VO reducing both glucose and oxygen.

Neuropathological changes that result from 2VO resemble those of late-onset AD, strengthening its use as an experimental model (de la Torre, 1999, 2000). Since hippocampal damage is a feature of AD, and the hippocampus is also particularly sensitive to ischemia, the time course of damage to the hippocampus as a result of 2VO surgery has been well characterized. While conspicuous hippocampal damage is not seen during the first week after 2VO (Ohtaki et al., 2006), damage to the CA1 subfield is observed in 6−29% of animals at 2 weeks (Schmidt-Kastner et al., 2001; Farkas et al., 2006), 55% at 4 weeks (Ohtaki et al., 2006), and total hippocampal destruction was observed in 67% of 2VO rats at 8−13 weeks (Farkas et al., 2004; Liu et al., 2006).

2VO also serves as a good model for cognitive aging and dementia because it not only results in global neurological changes, but also results in learning and memory impairment (Cada et al., 2000; de la Torre, 2000). It has been well established that experimental cerebral hypoperfusion compromises spatial learning in rats (Farkas and Luiten, 2001; Liu et al., 2005; Shang et al., 2005). Non-spatial object recognition deficits have also been observed 60 and 90 days following 2VO surgery (Sarti et al., 2002), but we did not observe deficits in odor recognition after 30 days (Auchter et al., 2014).

Methylene blue is a blue dye with neurometabolic enhancing properties at low doses (Rojas et al., 2012). MB crosses the blood-brain barrier and diffuses into the mitochondrial matrix, where at low doses it forms a redox equilibrium with the enzymes of the electron transport chain (Bruchey and Gonzalez-Lima, 2008; Riha et al., 2011). MB enhances mitochondrial respiration, chiefly by increasing the activity of cytochrome oxidase (CO) (Rojas et al., 2012). CO is the terminal enzyme of the electron transport chain and its activity reflects neuronal activity (Wong-Riley, 1989), including neuronal activity of circuits involved in rat water maze tasks (Villarreal et al., 2002; Conejo et al., 2010). MB enhancement of CO activity is coupled with increases in ATP production and oxygen consumption (Riha et al., 2005; Atamna et al., 2008; Bruchey and Gonzalez-Lima, 2008). Through enhanced mitochondrial efficiency, MB also reduces the incidence of reactive oxygen species formation (Salaris et al., 1991) and consequently delays cellular senescence (Atamna et al., 2008).

Methylene blue at low doses has been shown to enhance both spatial (Callaway et al., 2002; Riha et al., 2005; Wrubel et al., 2007) and non-spatial (Gonzalez-Lima and Bruchey, 2004) memories, and such enhancement is also marked by increases in CO activity (Callaway et al., 2002, 2004). In humans, MB enhances various memory tasks and modulates brain functional connectivity (Telch et al., 2014; Rodriguez et al., 2016, 2017; Zoellner et al., 2017). MB has also been shown to be neuroprotective in rodent models of stroke (Salaris et al., 1991; Miclescu et al., 2010; Shen et al., 2013), hypoxia (Huang et al., 2013), Alzheimer’s disease (Callaway et al., 2002; Riha et al., 2005), Parkinson’s disease (Rojas et al., 2009b; Smith et al., 2017), and mitochondrial optic neuropathy (Zhang et al., 2006; Rojas et al., 2009a). However, MB effects have not been described during chronic cerebral hypoperfusion.

The objectives of this experiment were to describe (1) how 2VO weakens brain regional cytochrome oxidase activity as measured by quantitative enzyme histochemistry, interregional correlation of cytochrome oxidase activity and performance on a visual discrimination task, (2) how MB strengthens performance and preserves both regional cytochrome oxidase activity and interregional correlation of cytochrome oxidase activity, and (3) how specific changes in brain regional cytochrome oxidase activity are correlated with behavioral performance (Auchter, 2015).

Our hypothesis was that chronic cerebral hypoperfusion results in reduction in regional CO activity in brain regions susceptible to hypoxia and a reduction in interregional correlation of cytochrome oxidase activity between regions involved in visual discrimination learning. Treatment of chronic cerebral hypoperfusion with daily low-dose methylene blue (MB) injections may restore both brain regional activity and interregional correlation of cytochrome oxidase activity, preventing neurodegeneration and memory impairment.

## Materials and Methods

### Subjects

Subjects for this brain analysis were the same subjects used in the behavioral study of Auchter et al. (2014). Subjects were 39 adult male Long-Evans rats weighing approximately 500−600 g at the time of surgery. Two rats died from surgical complications and one rat died of a respiratory infection during visual water task training. Additionally, it was determined upon brain extraction that one subject suffered a hemorrhagic stroke, and thus was excluded from the analysis, making the final *N* = 35.

### Ethical and Biosafety Measures

Rats were raised from birth in Association for Assessment and Accreditation of Laboratory Animal Care (AAALAC)-approved facilities under standard laboratory conditions (12 h: 12 h, light: dark cycle) with *ad libitum* access to food and water. All animal care and experimental procedures were approved by the University of Texas at Austin’s Institutional Animal Care and Use Committee (IACUC), and followed NIH guidelines as described in the Guide for the Care and Use of Laboratory Animals, 8th edition. All animal research was conducted in the Animal Resources Center at the University of Texas at Austin, an AAALAC-accredited facility which is inspected twice a year by UT’s IACUC to ensure compliance with all relevant laws, regulations, and policies regarding animal care and use. Personal protective equipment (PPE) such as lab coats, gloves, and face masks were used for all animal interactions. Cytochrome oxidase histochemistry was performed in the histochemical suite of the PI’s lab in the Animal Resources Center, which is inspected and approved twice a year by UT’s Environmental Health and Safety department. PPE was used during all stages of the histochemical staining procedure, and all chemical storage and usage complied with NIH guidelines as described in the NIH Chemical Safety Guide (2015).

### Surgical Procedures and Interventions

**FIGURE 1:**
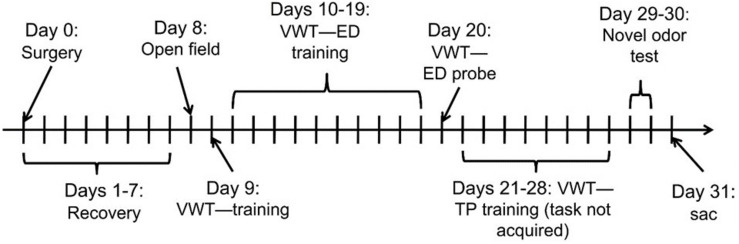
Experimental design and timeline. After 2VO or sham surgery (Day 0), rats received daily intraperitoneal injections of 4 mg/kg MB or saline (2 × 2 design = four groups). Rats recovered for 1 week (Days 1–7) before the start of behavioral training and testing (Days 8–30). VWT, visual water maze task; ED, elemental pattern discrimination; TP, transverse pattern discrimination; sac, sacrifice on Day 31 to collect brains for analysis.

Auchter et al. (2014) provides detailed explanations of the surgical and behavioral procedures, and the experimental design is shown in Figure 1. Briefly, the experiment followed a 2 × 2 experimental design, whereby subjects were randomly assigned to groups which were given either 2VO surgery or a sham control (without vessel occlusion), followed by daily intraperitoneal injections of either 4 mg/kg MB or an equivalent volume of saline.

Before surgery, we anesthetized the rats beginning with 4% isoflurane inhalation, followed by general anesthesia using 1.5−3% during the surgery, with an E-Z Anesthesia Vaporizer (Euthanex Corp., Palmer PA, United States). During the surgery, we made an incision to the midline of the neck, ventral aspect. We exposed the carotid arteries, dissecting them free from the sheath and from the vagal nerve as in de la Torre and Fortin (1991). For the 2VO group, we double-ligated each artery, posterior to the bifurcation of the carotid, with 4-0 silk sutures. For the sham group, we dissected the carotids free from the sheath, but did not proceed with the occlusion. Before closing, we injected each subject subcutaneously with 1 mg/kg of meloxicam (a surgical analgesic), as well as either MB or saline. Those subjects randomly assigned to the MB group were given the first intraperitoneal injection of MB (4 mg/kg body weight; USP methylene blue, Spectrum, New Brunswick, NJ, United States). Those subjects in the saline group received the same volume of physiological saline. We then sutured the incision and moved the subject to a cage for recovery, monitoring them for 30 min closely, before returning the subject to the home cage.

For the following 30 days, each subject in the MB group received one intraperitoneal injection per day of 4 mg/kg MB, dissolved in saline. The saline group received intraperitoneal injections of physiological saline vehicle for 30 days. The injections were timed to occur just after each daily training session, since there is evidence that methylene blue facilitates memory consolidation when given during the memory consolidation phase post-training (Martinez et al., 1978; Callaway et al., 2004). The subjects recovered for 1 week prior to behavioral testing.

### Visual Water Maze Task

Upon recovery from surgery, subjects were trained on the visual water task (VWT), as described in Prusky et al. (2000). The VWT using a water Y-maze is a variant of the Morris water maze, with some additional features. In the visual water Y-maze task, the subject must make a choice to commit to which arm contains the escape platform. In addition, explicit visual cues are given to the subject on which arm is correct/incorrect. This allows for a more complex experimental design than the standard Morris water maze, one that includes separate sets of “positive” (+ = approach) and “negative” (− = avoidance) visual stimuli. These visual stimuli involve visual patterns that are more similar to the visual discrimination tests given to humans to test cognitive function. In paradigms like the elemental discrimination task we used, these visual cues are progressively added to make the task progressively more difficult, which would not be possible in the standard Morris water maze. It is important for this study to make the maze task progressively more difficult to be able to reveal progressive memory deficits that develop over days after chronic 2VO surgery.

A metal water-filled trapezoid-shaped Y-maze tank, with one end composed of clear Plexiglas, was used for the task as described in Auchter et al. (2014). Stimuli were presented using a computer monitor, which was located on the other side of the transparent wall of the tank. A divider on the midline bisected the tank, extending from the transparent wall. The divider created two arms, with two different patterns visible on the monitor at each end. During each individual trial, a transparent platform was placed at one arm’s end. It was completely submerged and invisible to the subject. Each arm was paired with a visual pattern presented on the monitor. Randomization of the arms for each trial and recording of behavioral results were controlled by a computer program (Acumen; CerebralMechanics Inc.). A correct pattern (+) predicted which arm contained the escape platform. An incorrect pattern (−) predicted the arm without the platform.

During the first day of VWT training, the water tank contained an insert, which was placed such that the rat was forced to swim down one arm only (without the option of swimming down the other arm) for 15 trials. The escape platform was consistently placed at the end of the open arm, and the monitor was blank (no pattern was presented). The subject was placed in the tank, and swam around, discovering the platform at the end. After climbing onto the platform, each rat was removed and put into a heated recovery cage. Thus, the subjects were trained to associate swimming and reaching the platform with escape from the water-filled tank. This also allowed the observation of swimming ability, independent of the discrimination tasks to come. All of the subjects successfully learned the location of the platform at the end of the first few trials. By trial number 10, all of the subjects were consistently swimming straight to the escape platform immediately after they were placed in the water.

Visual water task protocols were adapted from those described by Driscoll et al. (2005). In the first variation of the VWT, subjects were trained for 10 days on three elemental pattern discriminations (A+B−, C+D−, and E+F−). Each subject is placed into the water at the opposite end of the water tank, facing the monitor. The subject swims into one of the arms, toward one of the stimuli presented onscreen. If the arm is correct, the subject climbs onto the escape platform. If the arm is incorrect (i.e., the escape platform is not present), a metal divider is used to block that arm for 10 s, then removed. The subject then swims into the correct arm to reach the escape platform. After reaching the platform, the subject is removed from the water and placed in a cage until the next trial begins. Subjects were trained on 30 trials per day in a stepwise manner: A+B− first, followed by intermixed trials of A+B− and C+D−, followed by intermixed trials of A+B−, C+D−, and E+F−. Every subject reached a criterion of 8/10 correct trials on the A+B− phase within 4 days. After 10 days, a set of 30 probe trials consisted of randomly alternated presentations of each of the three sets of visual stimuli (with 10 trials for each set). In the second variation of the task, subjects were trained on a more difficult transverse pattern discrimination (X+Y−, Y+Z−, and Z+X−).

### Quantitative Cytochrome Oxidase Histochemistry

Cytochrome oxidase brain mapping was conducted by an investigator blind to experimental condition through a well-established method of optical densitometry of brain sections as detailed by Gonzalez-Lima and Cada (1994). Following behavioral procedures, animals were sacrificed, and brains were quickly removed and frozen in isopentane. Coronal brain sections (40 μm thick) were obtained and mounted on slides using a cryostat microtome (Microm HM-505E, Heidelberg, Germany). To obtain increasing gradients of CO activity, a frozen brain homogenate paste was sectioned into different tissue thicknesses (10, 20, 40, 60, and 80 μm) and mounted on separate slides. CO activity of the homogenate was spectrophotometrically assessed and used to calculate CO values for the different section thicknesses. The brain paste sections were used as calibration standards for each CO staining batch.

Each CO staining batch consisted of representative cryostat sections (40 μm thick) from two brain levels mounted on microscope slides for each subject along with two slides with a set of 5 standards each. Each batch was processed for CO histochemistry following the method previously described by Gonzalez-Lima and Jones (1994). Briefly, slides were fixed for 5 min with 1.5% glutaraldehyde, rinsed three times in phosphate buffer with sucrose and preincubated in a solution containing cobalt chloride and DMSO dissolved in Tris buffer. After rinsing sections with phosphate buffer (pH 7.6; 0.1 M), they were incubated at 37°C for 1 h in the dark and with continuous stirring in a solution containing diaminobenzidine, sucrose, cytochrome c and catalase (Sigma-Aldrich, Barcelona, Spain) dissolved in phosphate buffer (pH 7.6; 0.1 M). The slides were then dehydrated in increasing concentrations of ethanol, coverslipped with permount (Merck, Darmstadt, Germany) and allowed to dry for 48 h.

To obtain CO activity values for regions of interest (ROIs), a calibration step tablet (Kodak, density range 0.06−3.05) and CO-stained slides were placed on a high-precision illuminator and digitized using a CCD digital microscope camera (Leica Microsystems DFC450, Wetzlar, Germany). Images were then analyzed using ImageJ software, which allows the creation of a logarithmic calibration curve of optical density units as a function of pixel (gray level) values in each image. Before analysis, images were corrected for slide and light box artifacts using background subtraction. For brain structure analyses, 44 ROIs were located and outlined in each brain hemisphere in each subject by an experimenter blind to experimental conditions using a rat brain atlas (Paxinos and Watson, 1996; see Table 1 for a complete list of ROIs and abbreviations). The measurement obtained was an average optical density (OD) value for the outlined ROI. To maximize the accuracy of OD values, this procedure was repeated in three adjacent brain sections for each ROI, and the mean of the three sections in both cerebral hemispheres was used as the overall mean OD value for that ROI. Mean OD values were later converted into mean CO activity units using calibration curves based on tissue standards and spectrophotometrically determined CO activity. This method yielded a linear relationship (*r* > 0.95) between biochemical CO activity measured spectrophotometrically and histochemical CO reactivity measured by optical density.

**TABLE 1:** Mean CO activity values (μmol/min/g) ± standard errors (SEM) for each ROI and each experimental condition.

ROI	Sham ± saline SEM	Sham ± MB SEM	2VO ± saline SEM	2VO ± MB SEM
Prelimbic cortex (PLC)	246.43 ± 9.88	243.80 ± 13.63	225.09 ± 16.21	247.76 ± 9.69
Medial orbital cortex (MO)	245.24 ± 11.49	239.99 ± 11.85	221.16 ± 15.31	248.61 ± 9.82
Ventral orbital cortex (VO)	254.38 ± 9.57	248.35 ± 12.49	235.53 ± 12.42	254.43 ± 8.99
Lateral orbital cortex (LO)	251.27 ± 8.88	249.62 ± 11.52	233.50 ± 11.32	252.09 ± 7.87
Agranular insular cortex (AI)	227.63 ± 7.31	220.18 ± 8.90	211.33 ± 10.82	229.76 ± 8.69
Infralimbic cortex (IL)	281.87 ± 9.58	270.41 ± 14.29	269.97 ± 15.16	272.44 ± 5.28
Primary motor cortex (M1)	237.17 ± 4.92	230.15 ± 10.02	222.54 ± 13.23	232.86 ± 3.74
Secondary motor cortex (M2)	242.28 ± 6.10	233.62 ± 10.50	**214.49** ± 8.99	243.12 ± 7.61
Primary somatosensory cortex (S1)	214.27 ± 13.65	214.25 ± 7.76	222.05 ± 11.91	212.94 ± 7.10
Secondary somatosensory cortex (S2)	226.35 ± 7.55	218.51 ± 10.34	210.28 ± 7.41	230.64 ± 8.22
Cingulate cortex (Cin)	259.93 ± 8.32	244.58 ± 10.16	258.77 ± 15.84	257.88 ± 9.85
Perirhinal cortex (PRh)	230.05 ± 10.87	210.98 ± 8.89	**186.46** ± 14.63	223.64 ± 10.99
Primary visual cortex (V1)	241.59 ± 10.36	242.43 ± 7.87	**201.47** ± 12.24	247.19 ± 9.66
Secondary visual cortex (V2)	249.72 ± 11.05	249.49 ± 9.86	**206.10** ± 13.52	**239.80** ± 7.90
Medial septal nucleus (MS)	203.40 ± 8.33	197.97 ± 12.39	202.64 ± 10.36	197.97 ± 6.04
Lateral septal nucleus (LS)	272.30 ± 8.28	262.01 ± 12.81	273.92 ± 11.81	268.67 ± 5.58
Acumbens core (AcbC)	313.40 ± 9.76	304.24 ± 10.43	315.15 ± 17.36	307.88 ± 7.59
Acumbens shell (AcbS)	241.13 ± 10.53	223.67 ± 8.36	233.98 ± 8.35	224.90 ± 7.96
Caudate/Putamen (CPu)	198.87 ± 13.32	199.51 ± 8.52	191.14 ± 11.26	191.27 ± 6.12
Globus palidus (GP)	115.97 ± 6.57	108.53 ± 4.83	115.23 ± 5.74	112.64 ± 4.92
Ventral pallidum (VP)	179.11 ± 5.82	169.26 ± 6.40	168.38 ± 8.74	168.31 ± 4.50
Lateral hypothalamus (LH)	160.75 ± 9.32	157.53 ± 9.78	154.51 ± 5.90	163.81 ± 8.06
Paraventricular nucleus (PVH)	191.74 ± 6.94	183.50 ± 8.14	184.33 ± 8.43	203.21 ± 6.86
Medial preoptic area (MPO)	206.22 ± 6.62	198.81 ± 7.51	210.35 ± 10.84	208.42 ± 5.72
Bed nucleus stria terminalis (BST)	233.80 ± 7.98	218.64 ± 9.49	237.61 ± 14.51	224.12 ± 6.61
Subthalamic nucleus (Sub)	273.20 ± 6.04	249.10 ± 10.74	249.36 ± 14.37	248.75 ± 10.91
Medial geniculate nucleus (MGN)	255.28 ± 8.38	245.48 ± 5.66	240.78 ± 12.86	235.70 ± 5.45
Basomedial amygdala (BMA)	186.21 ± 7.73	180.59 ± 10.02	175.09 ± 4.88	195.70 ± 9.16
Medial amygdala (MEA)	227.95 ± 10.39	220.31 ± 11.47	**194.29** ± 9.88	226.00 ± 9.75
Basolateral amygdala (BLA)	268.46 ± 14.51	254.36 ± 14.44	**230.77** ± 12.21	266.47 ± 12.53
Central amygdala (CEA)	223.13 ± 8.63	219.37 ± 10.03	206.30 ± 9.63	234.55 ± 9.36
Anterior hippocampus CA1 (CA1A)	213.48 ± 11.42	199.98 ± 8.20	**188.32** ± 18.91	213.97 ± 13.79
Anterior hippocampus CA2 (CA2A)	224.26 ± 13.21	203.44 ± 8.69	**195.70** ± 16.03	214.87 ± 11.19
Anterior hippocampus CA3 (CA3A)	238.88 ± 15.71	213.68 ± 10.05	**200.94** ± 15.59	216.38 ± 8.13
Anterior dentate gyrus (DGA)	319.76 ± 26.01	294.90 ± 15.21	306.25 ± 31.40	297.03 ± 19.93
Posterior hippocampus CA1 (CA1P)	298.13 ± 13.98	277.27 ± 12.72	266.56 ± 14.28	277.08 ± 13.51
Posterior hippocampus CA2 (CA2P)	275.84 ± 11.87	249.69 ± 11.82	236.40 ± 17.96	248.44 ± 10.82
Posterior hippocampus CA3 (CA3P)	292.89 ± 9.91	272.22 ± 13.02	255.42 ± 16.45	269.08 ± 9.99
Posterior dentate gyrus (DGP)	269.60 ± 15.75	256.92 ± 10.66	258.72 ± 12.20	255.88 ± 7.02
Subiculum (S)	239.98 ± 12.04	226.65 ± 10.53	221.74 ± 11.20	217.73 ± 6.72
Superior colliculus (SC)	254.18 ± 13.53	246.46 ± 8.80	240.89 ± 10.46	249.47 ± 8.05
Substantia nigra (SN)	214.80 ± 8.68	207.29 ± 8.24	211.66 ± 11.57	209.81 ± 6.72
Red nucleus (R)	250.37 ± 12.02	251.90 ± 13.05	234.69 ± 13.86	257.29 ± 12.34
Ventral tegmental area (VTA)	97.29 ± 1.32	99.91 ± 5.86	88.06 ± 3.55	96.05 ± 5.11

*Means showing an individual group effect are boldfaced.*

Figure 7 shows how the ROI was outlined for CO measures. For regional CO activity measures, when 2VO-induced lesions were present within the ROI, both the affected and surviving tissue were included in the outlined ROI (i.e., the ROI was defined using the brain atlas, not the lesion). The reason for doing this was two-fold. First, it allowed us to index general CO activity in each ROI, taking into account both lesions and compensatory increases in CO in surviving tissue (if such compensatory phenomena existed). Second, since some lesions did not have clearly defined borders, including the entire ROI gave us measures of CO activity that were unbiased by experimenter evaluation of lesioned vs. penumbra vs. healthy tissue.

### Lesion Volume Analysis

An assessment of lesion volume was conducted in a separate analysis. In 2VO subjects that showed lesions (*n* = 6), volumetric analysis was conducted on affected areas. Lesion volumes (V) were derived from the lesion area per slice (A) and distance between collected sections (d), using the formula V = ΣA × d. The value d was calculated as *d* = (T) × (number of designated sections-1), where T is the distance between every designated section (80 μm). Lesion area per slice was measured by setting a conservative optical density threshold aimed to prevent the inclusion of any white matter and tissue artifacts as affected tissue. This was especially important since we observed lesions in the striatum, which in rats is speckled with white matter tracts. To ensure the threshold was unbiased, for each subject the minimum and maximum threshold values were set to 85% and 98% of the measured mean OD value for white matter (i.e., “more OD” than white matter, but “less OD” than healthy gray matter tissue). A few subjects showed tissue loss where lesions occurred. In these cases, the maximum value for the threshold was set equal to white matter so that regions with missing tissue were still considered in the calculation of lesion area.

### Statistical Analyses

All statistical testing was carried out using the PASW 22 software package (SPSS, Inc., Chicago, IL, United States). All differences were considered statistically significant at the two-tailed *p* < 0.05 level. Effect sizes were estimated using Cohen’s d (Cohen, 1988). For univariate group comparisons, average CO values for each ROI were used as dependent variables in separate analyses, with surgery and drug treatment as independent variables.

Since subjects went through two iterations of the visual water task (elemental discrimination and transverse pattern discrimination), a composite “VWT performance score” was calculated by averaging the scores from the final probe for each task. Though subjects did not learn the transverse task (as indicated by the inability to reach and 8/10 criteria for pattern discriminations), their performance in the task likely still influenced CO activity. Thus, brain-behavior correlations were conducted using two-tailed bivariate correlations between the VWT performance score and mean CO activity for each ROI.

Interregional correlation of cytochrome oxidase activity was analyzed in two ways. First, two-tailed bivariate correlations were performed between all brain ROIs within each experimental condition. Then, to detect which regional correlations showed significant differences between groups, correlation coefficients were compared using Fisher’s z-transformations, followed by independent samples *t*-tests (Vélez-Hernández et al., 2014).

## Results

The results were obtained by comparisons between four groups (2 × 2 design): sham + saline group, sham + MB group, 2VO + saline group, and 2VO + MB group. Since the experimental design was the same as in Auchter et al. (2014), detailed explanations of behavioral procedures (and behavioral results) can be found there, and an experimental timeline is shown in Figure 1.

### Region of Interest (ROI) Analysis

**FIGURE 2:**
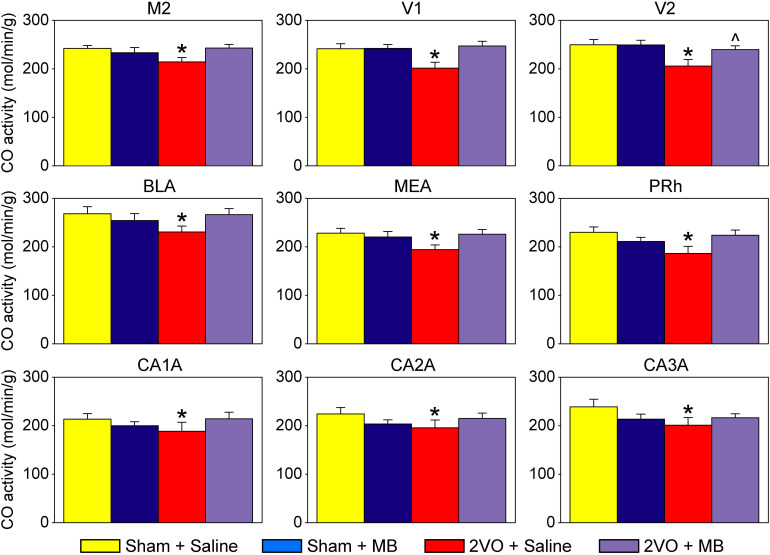
Regions of interests showing significant effects. In these regions, 2VO surgery (red) resulted in a decrease in cytochrome oxidase activity, which was prevented by MB treatment (violet). M2, secondary motor cortex; V1, primary visual cortex; V2, secondary visual cortex; BLA, basolateral amygdala; MEA, medial amygdala; PRh, perirhinal cortex; CA1A, anterior hippocampus CA1; CA2A, anterior hippocampus CA2; CA3A, anterior hippocampus CA3. *Significant surgery by drug interaction (*p* < 0.05); ^Significant main effect of surgery (*p* < 0.05).

Table 1 shows mean CO values and standard errors for each ROI in each experimental condition. Only the secondary visual cortex (V2) showed a significant main effect of surgery, *F*(1,31) = 4.491, *p* = 0.047, with an effect size (Cohen’s d) of 0.82, indicating a large effect size, given that | d| < 0.2 = small effect; 0.2 < | d| < 0.8 = medium effect; | d| > 0.8 = large effect (Cohen, 1988). There were no main effects for drug treatment. Several regions showed significant surgery by drug treatment interactions, including perirhinal cortex, *F*(1,31) = 5.667, *p* = 0.024, Cohen’s d = 0.81; secondary motor cortex, *F*(1,31) = 5.883, *p* = 0.022, Cohen’s d = 0.73; primary visual cortex, *F*(1,31) = 4.606, *p* = 0.045, Cohen’s d = 0.74; basolateral amygdala, *F*(1,31) = 5.350, *p* = 0.028, Cohen’s d = 0.61; medial amygdala, *F*(1,31) = 5.189, *p* = 0.030, Cohen’s d = 0.63; and the anterior portions of the hippocampus, subfields CA1, *F*(1,31) = 4.951, *p* = 0.034, Cohen’s d = 0.48; subfield CA2, *F*(1,31) = 4.650, *p* = 0.039, Cohen’s d = 0.53; and subfield CA3, *F*(1,31) = 4.940, *p* = 0.034, Cohen’s d = 0.53. These significant surgery by drug treatment interactions are shown in Figure 2.

### Interregional Correlation of Cytochrome Oxidase Activity

**FIGURE 3:**
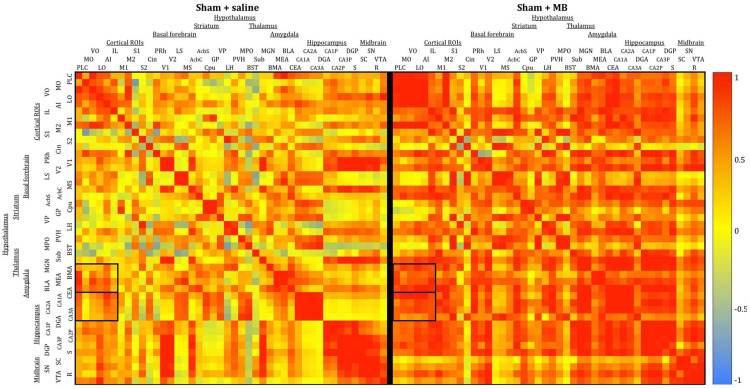
Heat maps showing interregional correlations for the sham + saline group **(left)** and sham + MB group **(right)**. The purpose of these diagrams is to show the differences in the overall pattern of correlation, rather than individual pairs. Colors correspond to Pearson’s r correlation coefficients, with red indicating strong positive correlation and blue indicating strong negative correlations. Generally, the MB group showed a greater number of positive correlations (red) than the saline group. Black boxes outline correlations between prefrontal ROIs and amygdala ROIs and prefrontal ROIs and hippocampal ROIs.

**FIGURE 4:**
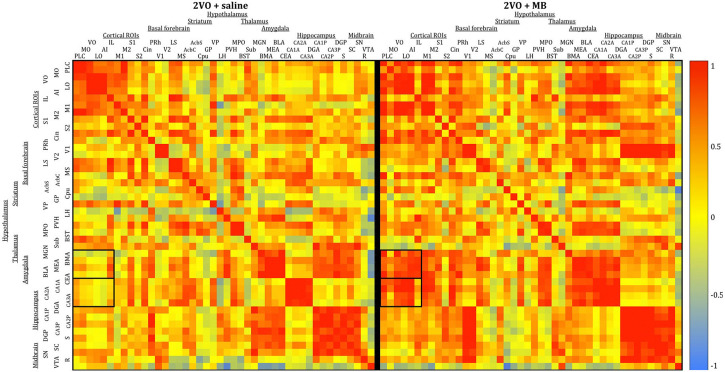
Heat maps showing interregional correlations for the 2VO + saline group **(left)** and 2VO + MB group **(right)**. Colors correspond to Pearson’s r correlation coefficients, with red indicating strong positive correlations and blue indicating strong negative correlations. Black boxes outline correlations between prefrontal ROIs and amygdala ROIs and prefrontal ROIs and hippocampal ROIs.

**FIGURE 5:**
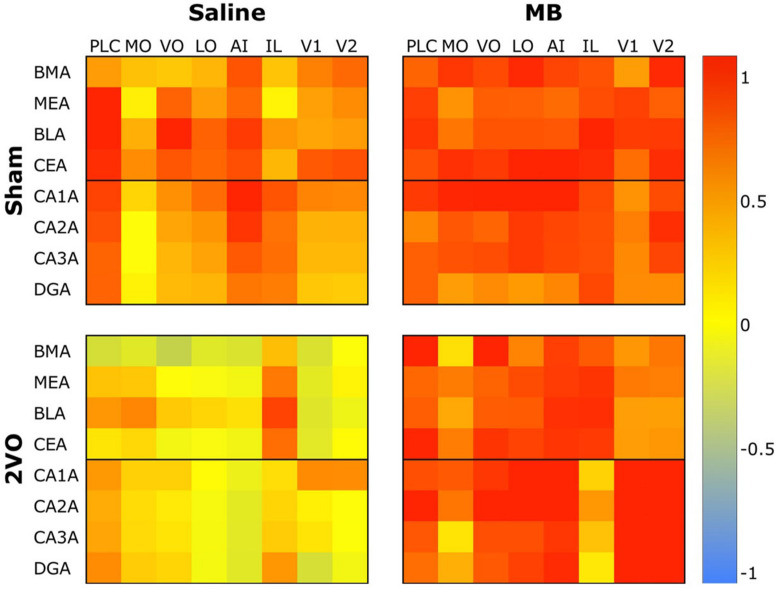
Heat map comparing interregional correlation of cytochrome oxidase activity between prefrontal ROIs and amygdala and hippocampal ROIs in each experimental condition. Colors correspond to Pearson’s r correlation coefficients, with red indicating strong positive correlations and blue indicating strong negative correlations.

To analyze interregional correlation of cytochrome oxidase activity, CO values for each ROI were correlated with each other for each group. Functional heat maps for these correlation matrices are shown in Figures 3, 4. Upon visual comparison of these maps, one can qualitatively deduce that MB treatment generally increased interregional correlation of cytochrome oxidase activity, specifically in a positive direction (i.e., MB resulted in more “redness” in the heat maps). This is true particularly in the sham condition (Figure 3). The result of MB treatment in the 2VO condition is not as obvious, though still apparent (Figure 4). One can also compare the left sides of Figures 3, 4 to observe an apparent decrease in interregional correlation of cytochrome oxidase activity as a result of 2VO surgery. The most dramatic differences in interregional correlation of cytochrome oxidase activity appear in the outlined boxes in Figures 3, 4 (also represented in larger form in Figure 5), which represent connectivity between prefrontal and visual cortex ROIs, and amygdala and hippocampal ROIs. Figure 5 shows stronger interregional correlation of cytochrome oxidase activity in the sham animals given MB as compared to saline. Importantly, it appears that the connectivity between cortical, amygdala and hippocampal ROIs is decreased in subjects that received 2VO, but connectivity is restored in 2VO subjects treated with MB.

**FIGURE 6:**
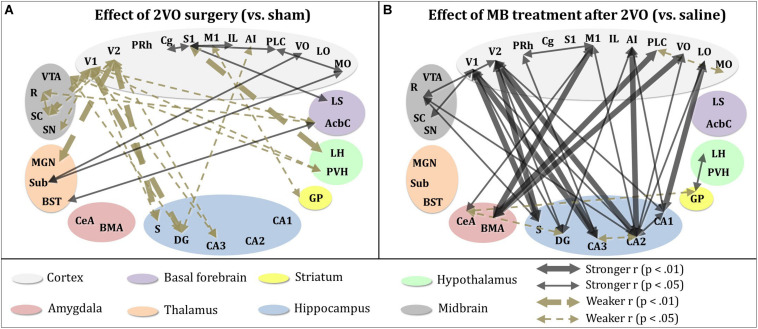
Significant differences in interregional correlation of cytochrome oxidase activity due to 2VO surgery and MB treatment. **(A)** Comparison between control subjects (sham + saline group) and 2VO subjects (2VO + saline). 2VO surgery resulted in weaker interregional correlation of cytochrome oxidase activity (1) within ROIs involved in the visual pathway (V1, V2, SC), and (2) between visual ROIs and hippocampus (CA3, DG, S). Stronger interregional interregional correlation of cytochrome oxidase activity was observed within cortical ROIs (MO, VO, PLC, IL, S1, Cg). **(B)** Comparison between 2VO subjects treated with saline and those treated with MB. In 2VO subjects, treatment with MB resulted in stronger interregional correlation of cytochrome oxidase activity (1) within ROIs involved in the visual pathway (V1, V2, SC), (2) between visual ROIs and hippocampus (CA1, CA2, CA3, DG, S), (3) between cortical ROIs (LO, VO, AI, M1) and hippocampus (CA1, CA2, DG), and (4) between cortical ROIs (VO, PLC, M1) and amygdala (BMA, CeA). Mildly weaker interregional correlation of cytochrome oxidase activity was observed between the amygdala (CeA), hippocampus (DG), and striatum (GP).

To further explore the effects of cerebral hypoperfusion and MB treatment on interregional correlation of cytochrome oxidase activity, bivariate correlation comparisons for each ROI were conducted (1) between the sham + saline group (control subjects) and 2VO + saline group, and (2) between the 2VO + saline group and the 2VO + MB group (Figure 6). The first group of comparisons essentially reveals which interregional correlations were significantly strengthened or weakened as a result of cerebral hypoperfusion. The second group of comparisons reveals which interregional correlations were significantly strengthened or weakened as a result of MB treatment during cerebral hypoperfusion.

**FIGURE 7:**
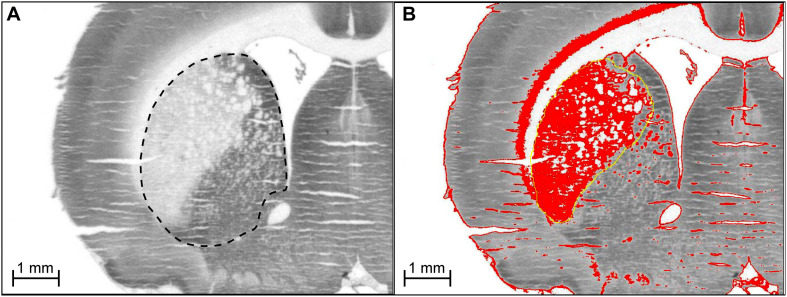
**(A)** Delineation of region of interest for CO measurement. For regional CO activity measures, when 2VO-induced lesions were present within the ROI, both the affected and surviving tissue were included in the outlined ROI (i.e., the ROI was defined using the brain atlas, not the lesion). **(B)** Determination of 2VO-induced lesion volume in rat brain. For every coronal section showing a lesion (e.g., lateral side of striatum), a threshold was applied to highlight (in red) tissue with optical density (OD) values between that of white matter and healthy gray matter tissue (medial side of striatum). For each section showing a lesion, the area of the highlighted tissue within the approximate lesion outline (in yellow) was used as A in the equation to calculate lesion volume, V = ΣA × d, where d is the distance between sections. The sections were stained for CO activity by the quantitative enzyme histochemistry method of Gonzalez-Lima and Jones (1994). We are showing the actual digitized images we used for the densitometric quantification of CO activity. Microphotographs with high contrast are not suitable for demonstration of a linear staining reaction product resulting from graded CO enzymatic activity as explained in our book on quantitative CO enzyme histochemistry with more details on this topic (Gonzalez-Lima, 1998). Briefly, digitized images with low contrast showing a linear gray level range are necessary to perform densitometric computer analysis of quantitative differences in CO activity from histochemically stained frozen brain sections and CO activity standards.

As shown in the left panel of Figure 6, interregional correlation of cytochrome oxidase activity between visual cortices and basal forebrain, hippocampal and midbrain ROIs was weakened as a result of cerebral hypoperfusion. However, interregional correlation of cytochrome oxidase activity between cortical regions—particularly between secondary somatosensory cortex and prefrontal ROIs—was strengthened as a result of cerebral hypoperfusion. This could potentially be evidence of a compensatory mechanism activated in response to the functional disconnection between regions involved in the visual network.

As shown in the right panel of Figure 6, interregional correlation of cytochrome oxidase activity between visual cortices and hippocampal ROIs—which was weakened in 2VO subjects—was strengthened as a result of MB treatment. Additionally, MB treatment resulted in increased interregional correlation of cytochrome oxidase activity between amygdala and cortical ROIs, and in midbrain ROIs with visual cortices and hippocampus.

### Neurodegenerative Lesions

**FIGURE 8:**
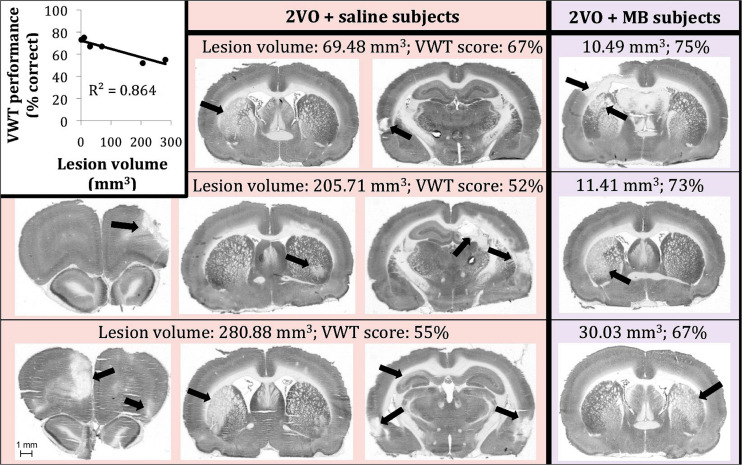
Six 2VO subjects showed neurodegenerative lesions (*n* = 3 saline-treated [pink background], *n* = 3 MB-treated [purple background]). There was a negative linear correlation between lesion size and VWT performance (top left). We are showing actual examples of digitized images of CO-stained coronal sections used for the determination of lesion volume.

**FIGURE 9:**
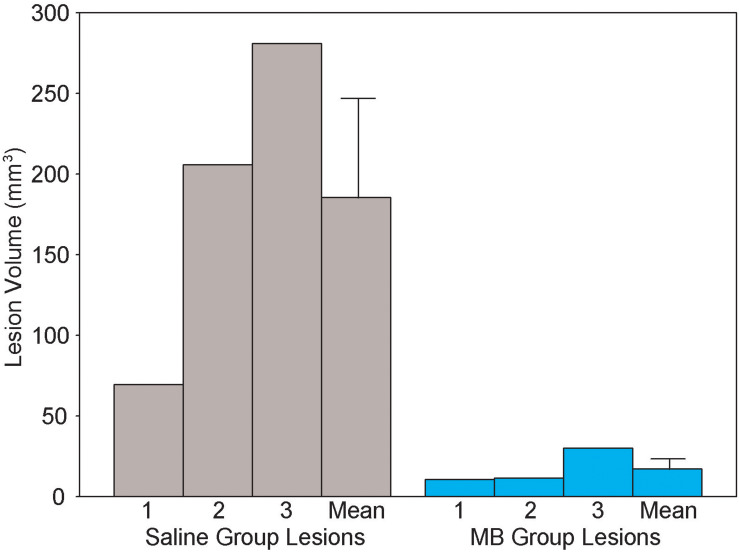
Lesion volume (cubic centimeters) in each of the six 2VO subjects which showed neurodegenerative lesions. Lesions were observed in *n* = 3 MB group subjects and *n* = 3 saline group subjects. Methylene blue treatment after 2VO surgery reduced the size of the lesions. Bars on means are standard errors.

Figure 7 illustrates the procedure for obtaining a conservative measure of neurodegenerative lesion area per section. About one third of subjects who received 2VO surgery (*n* = 3/9 in the 2VO + saline group and *n* = 3/8 in the 2VO + MB group) showed conspicuous anatomical lesions (Figure 8). Though there was quite a bit of heterogeneity in lesion size between subjects, the location of the lesions were categorically delimited, and thus were classified into 4 types: neocortical (occurring unilaterally or bilaterally in prefrontal regions and extending into primary motor, somatosensory and visual cortices; *n* = 2), striatal (occurring unilaterally in the caudate and putamen; *n* = 6), hippocampal (occurring unilaterally through the rostrocaudal extent of CA1, CA2 and CA3 subfields; *n* = 2), and perirhinal (occurring unilaterally or bilaterally in perirhinal cortex; *n* = 3). Qualitative and quantitative lesion comparisons revealed several interesting phenomena. First, there was a negative linear correlation between lesion size and VWT performance, indicating that the greater the overall lesion volume, the worse subjects performed on the VWT (Figure 8). Second, though the number of subjects displaying lesions is small, it appears that the emergence of the lesions followed a specific pattern. In subjects with the smallest lesion volumes, lesions were limited to striatum. In more severe cases, neocortical and perirhinal lesions appeared (Figure 8). Subjects with the largest lesion volumes showed striatal, neocortical, perirhinal and hippocampal lesions. Finally, though both carotid arteries were ligated in the 2VO surgery, when subjects had smaller lesions, the lesions were limited to either the right or the left cerebral hemisphere. Bilateral lesions only appeared in the most severe cases (in which subjects showed lesions in multiple locations, including hippocampus). Perhaps the most interesting observation in subjects displaying lesions was the impact of MB treatment. Though the same number of subjects showed lesions in each 2VO group, lesions in subjects treated with MB were smaller and more localized (i.e., only unilateral and only in/near striatum) than lesions in saline-treated 2VO subjects. The lesion volumes for each of the six subjects (as well as treatment group means) are shown in Figure 9.

### Brain−Behavior Correlations

**FIGURE 10:**
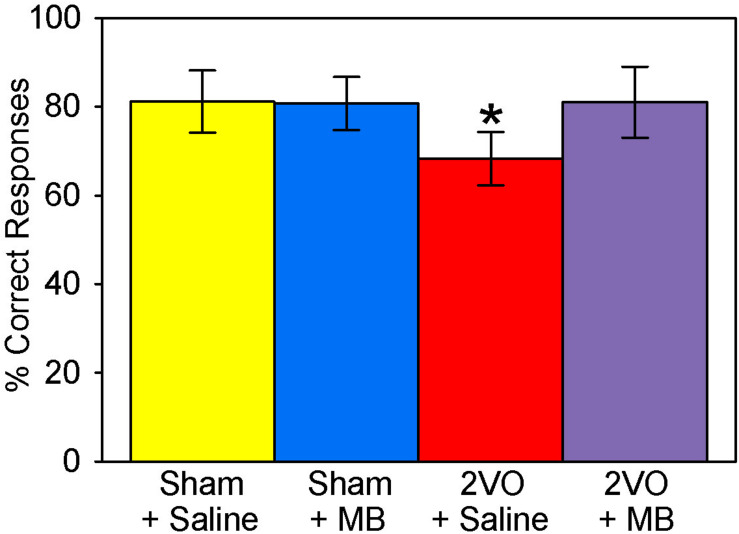
Visual water task performance scores. 2VO surgery resulted in worse overall scores on the VWT. However, 2VO subjects treated with MB performed no different from sham groups; *p < 0.05.

Like the behavior performance for the elemental discrimination alone (Auchter et al., 2014), a univariate ANOVA—with the composite VWT performance score as the dependent variable and the surgery and drug treatment conditions as independent variables—revealed a surgery by drug treatment interaction, *F*(1,31) = 8.961, *p* = 0.006. *Post hoc* pairwise comparisons demonstrated that subjects who received 2VO surgery performed worse than sham subjects (decreased percent correct responses), but this was ameliorated by MB treatment (Figure 10).

Visual water task performance scores were correlated with CO activity for a number of individual ROIs. Regions that showed significant positive brain-behavior correlations were perirhinal cortex, *r*(35) = 0.526, *p* = 0.001, prelimbic cortex, *r*(35) = 0.353, *p* = 0.038, secondary motor cortex, *r*(35) = 0.461, *p* = 0.005, primary visual cortex, *r*(35) = 0.432, *p* = 0.035, secondary visual cortex, *r*(35) = 0.410, *p* = 0.047, caudate/putamen *r*(35) = 0.343, *p* = 0.043, medial amygdala, *r*(35) = 0.368, *p* = 0.030, anterior CA1, *r*(35) = 0.455, *p* = 0.006, anterior CA2, *r*(35) = 0.390, *p* = 0.021, and anterior CA3, *r*(35) = 0.435, *p* = 0.009. There were no significant negative correlations between CO activity and VWT performance.

To assess whether correlations between regional CO activities and VWT performance were specifically driven by subjects showing lesions, brain-behavior correlations were also conducted excluding subjects who displayed lesions. When lesioned subjects were excluded, perirhinal cortex, *r*(29) = 0.397, *p* = 0.033, secondary motor cortex, *r*(29) = 0.409, *p* = 0.027, medial amygdala, *r*(29) = 0.424, *p* = 0.022, primary visual cortex, *r*(29) = 0.533, *p* = 0.0323, and secondary visual cortex, *r*(29) = 0.471, *p* = 0.049 still showed significant positive correlations between CO activity and behavioral performance. Hippocampal regions, prelimbic cortex and caudate/putamen no longer showed significant correlations. Interestingly, significant correlations emerged in agranular insular cortex, *r*(29) = 0.392, *p* = 0.035, and lateral orbital cortex, *r*(29) = 0.435, *p* = 0.018, when lesioned subjects were excluded. That many correlations persisted when lesions were absent suggests that the lesions are not entirely responsible for the observed brain-behavior correlations. However, correlations were not completely independent of lesions either, since some correlations disappeared when lesioned subjects were excluded. Further, since additional correlations appeared in non-lesions subjects (in ROIs that did not show lesions in lesioned subjects), the functional impact of lesions extended beyond the lesioned areas themselves.

## Discussion

Brain cells rely on the circulatory system to supply the necessary glucose and oxygen needed for mitochondrial respiration, oxidative phosphorylation and ATP synthesis (Erecinska and Silver, 1989). Cytochrome oxidase (CO)−a transmembrane protein localized in the inner mitochondrial membrane—is the terminal enzyme of the electron transport chain (Wong-Riley, 1989). It is well-known that chronic cerebral hypoperfusion results in impaired ATP synthesis (Plaschke, 2005; Brīede and Duburs, 2007), mitochondrial dysfunction and a reduction in CO activity (de la Torre et al., 1997; Cada et al., 2000). There is also evidence that this mitochondrial dysfunction is paralleled by deficits in learning and memory (de la Torre et al., 1997; Cada et al., 2000). Here, we used a model of chronic cerebral hypoperfusion, bilateral carotid occlusion (2VO), the neuropathological effects of which have been well-characterized and resemble those of age-related progression of mild cognitive impairment (MCI) and Alzheimer’s disease (AD) (de la Torre, 2016). We aimed to: (1) characterize the whole-brain regional and interregional correlation of cytochrome oxidase activity changes that result from 2VO surgery, (2) describe how these changes are ameliorated via treatment with the neurometabolic enhancer USP methylene blue (MB), and (3) correlate brain CO activity with performance on the visual water task (VWT).

### Regional Changes in Cytochrome Oxidase Activity

Regional changes in cytochrome oxidase activity as revealed by univariate analyses for each ROI were observed in cortical regions (secondary motor cortex, perirhinal cortex, primary visual cortex and secondary visual cortex), amygdala (basolateral and medial) and hippocampus (the anterior portions of CA1, CA2, and CA3). Though the neuropathological changes in hippocampus (Shang et al., 2005), retina (Lavinsky et al., 2006), and optic tract (Farkas et al., 2004) as a result of 2VO have been documented, regional differences in cortical areas specific to visually guided movements have only been documented one other time (Cada et al., 2000); and regional differences in the amygdala are novel findings.

Additionally, we show here that chronic cerebral hypoperfusion can affect regional cytochrome oxidase activity in two ways. First, regions susceptible to hypoperfusion show decreases in CO activity without developing anatomical lesions. This is evidenced by the fact that we observed univariate differences in CO activity as a result of 2VO surgery in regions that did not show lesions (e.g., in basolateral and medial amygdala). Interregional correlation of cytochrome oxidase activity changes observed in response to 2VO surgery in regions that do not show anatomical lesions further support this notion. Second, in about one third of subjects, 2VO surgery resulted in lesions, as indicated by absence of histochemical CO staining and presence of tissue damage. Studies comparing the CO method and other histological methods show that CO activity is a good proxy for cell density (Nelson and Silverstein, 1994; Wong-Riley and Welt, 1980; Riha et al., 2005). Thus we can infer that regions showing complete absence of CO activity in discrete identifiable regions are in fact damaged. Since this happens in less than half of the subjects and that lesion sizes vary so widely suggests that subjects are differentially susceptible to developing lesions during cerebral hypoperfusion after 2VO. This differential susceptibility not only results in variable lesion sizes, but greater lesion sizes are also highly correlated to impaired behavioral performance in the VWT.

Additionally, that lesions appear in regions where CO activity is particularly involved in visual discrimination learning (Fidalgo et al., 2014)−and that CO activity in these regions were also correlated with VWT performance−brings about the question of whether the lesions caused the behavioral deficits, or whether the intensive behavioral training facilitated the lesions. In one likely scenario, we see the appearance of lesions as a result of certain regions being particularly vulnerable to cerebral hypoperfusion. The lesions would then lead to an inability of these subjects to master the VWT task. However, in an alternative scenario, the VWT itself may have resulted in increased CO activity demand in particular regions preceding neurodegeneration. In this case, cerebral hypoperfusion could result in increased oxidative stress in regions involved in learning the VWT task, and thus would result in “customized” lesions in brain regions involved in a particular task. Though both scenarios may have contributed in some way to the appearance of lesions, we believe that the former scenario is the most plausible, primarily because similar regions have been previously identified as susceptible to cerebrovascular ischemia. Additionally, since CO activity in the medial amygdala is correlated with VWT performance, if lesions resulted from the demanding VWT training, then we should also expect to see amygdala lesions, which we did not. The potential emergence of task-specific lesions can be further addressed in future investigations by (1) comparing mitochondrial lesions in trained vs. untrained subjects experiencing cerebral hypoperfusion, and/or (2) training subjects on a different task involving different brain regions (e.g., an auditory rather than visual task), and exploring whether the lesion location changes (e.g., to auditory rather than visual cortices).

### Interregional Correlations of Cytochrome Oxidase Activity

2-vessel occlusion surgery resulted in weakened interregional correlation of cytochrome oxidase activity in several regions. The most obvious decoupling occurred between visual cortices (primary and secondary) and hippocampus (CA3, dentate and subiculum). 2VO subjects also showed weaker interregional correlation of cytochrome oxidase activity between visual cortex and the superior colliculus, further implicating 2VO surgery in the pathogenesis of the visual system. There were also some noteworthy increases in interregional correlation of cytochrome oxidase activity, namely between the subthalamic nucleus and prefrontal cortex (ventral and medial orbital cortex), between the bed nucleus of the stria terminalis and nucleus accumbens, and between several cortical regions (medial orbital cortex, prelimbic cortex, infralimbic cortex, cingulate, and primary somatosensory cortex). Because these increases in interregional correlation of cytochrome oxidase activity seemed to appear mostly in regions that were not significantly affected by 2VO surgery (as indicated by univariate analyses), this strengthened interregional correlation of cytochrome oxidase activity may indicate a compensatory mechanism that occurred in response to the weakened interregional correlation of cytochrome oxidase activity in visual and hippocampal regions. There is some evidence of compensatory mechanisms in 2VO, however most of this evidence concerns global normalization of cerebral blood flow via vascular changes, such as collateral perfusion and angiogenesis (Choy et al., 2006). Therefore, there is the possibility that MB may have preserved CO activity and memory by partly alleviating hypoperfusion in the ischemic brain, e.g., by increasing blood supply via the vertebral arteries after occluding the carotid arteries. Our own fMRI studies of cerebral blood flow indicate that MB can enhance blood flow under hypoxic conditions (Huang et al., 2013). To verify the existence of compensatory mechanisms in blood flow and metabolic activity, further investigation is needed.

### Biological Underpinnings of the Interregional Correlations of Cytochrome Oxidase Activity

At the cellular level, CO enzymatic activity reflects neuronal synaptic activity because CO is a rate-limiting enzyme in cellular respiration for ATP production, and ATP is required to sustain neuronal electrophysiological responses (Wong-Riley, 1989). Specifically, neuronal membranes depolarized by synaptic excitatory transmitters require ATP for repolarization to maintain electrophysiological responses. Moreover, at the transcriptional molecular level, there is a coupling of genes for CO enzymatic activity with genes for neuronal synaptic excitation (Wong-Riley, 2012). Specifically, the transcriptional regulation of CO genes is coupled with the transcription of nuclear respiratory factor genes, such as NRF-1 and NRF-2, which in turn are coupled with transcription of excitatory neurotransmitter receptors, such as NMDA receptor subunit genes for glutamatergic excitatory synaptic activity (GluN1 and GluN2) (Dhar and Wong-Riley, 2009; Wong-Riley, 2012; Nair and Wong-Riley, 2016). Therefore, all ten nuclear CO genes and the three mitochondrial CO transcription factors are transcribed in the same “transcription factory” that is neuronal activity-dependent (Wong-Riley, 2012). This makes quantitative CO histochemistry of individual brain regions an important metabolic index of neuronal activity (Gonzalez-Lima, 1998). Specifically, CO histochemical activity after 2VO provides insight into the metabolic history of brain regions during chronic brain hypoperfusion, as brain regions that are progressively less active will show reduced CO activity relative to regions with preserved neuronal activity (Cada et al., 2000).

Metabolic mapping techniques like quantitative CO histochemistry make it possible to gather functional data from most brain regions. However, most analyses of metabolic mapping data are limited to the comparison of mean regional activity between groups. This is like treating each brain region as if it were separate from the rest of the brain. However, the interactions between regions likely affect higher-order neurobiological functions underlying neural network communications and associative learning functions (Gonzalez-Lima and McIntosh, 1995). Differences in *regional* mean CO activity after chronic 2VO reflect differences in the cumulative neuronal activity of specific brain regions, whereas differences in *interregional* correlations of CO activity reflect cumulative differences in functional synaptic coupling among brain regions. At the interregional physiological level, CO activity is critical for the coordinated functioning of brain regions because aerobic cellular respiration is the main way neurons obtain metabolic energy to communicate via synapses. Hence, interregional correlations of CO activity represent a functional connectivity index of the metabolic history of interactive neuronal synaptic activity among brain regions (Gonzalez-Lima, 1998). However, the biological underpinnings of CO interregional correlations are very different from other metabolic markers such as uptake of 2-deoxyglucose or fluorodeoxyglucose, or c-fos gene/protein expression, all of which reflect evoked or immediate neuronal activity. As explained in detail by Sakata et al. (2000), interregional correlations of CO activity represent functional “traits” rather than an acute “state.”

In our study, differences in the magnitude of interregional CO correlations after chronic 2VO and MB reflected the metabolic history of functional synaptic relationships between brain regions that were part of specific neural networks. Although interregional CO effects need to happen via anatomical synaptic connectivity, anatomical and functional connectivity are not synonymous. For example, two regions may be similarly connected anatomically in the saline groups and the MB groups, but differences in their interactive synaptic activity would lead to different interregional correlations of CO activity. Specifically, we found different interregional correlations in CO activity among prefrontal and limbic brain regions within groups of subjects treated with saline or MB from sham and 2VO groups. We then established the patterns of statistical significance for each condition of treatment (saline/MB) and surgical (sham/2VO) groups and interpreted these results as differences in the metabolic history of functional synaptic connections between brain regions due to each condition.

Horwitz et al. (1992) reviewed the first studies that introduced the analysis of functional network interactions using interregional correlations of metabolic mapping data in the 1980s. This network approach became popular to analyze the relationships of blood oxygen level dependent (BOLD) signals from multiple brain regions in fMRI, and Friston (1994) called it functional connectivity analysis. The only difference in these analytic techniques is the type of metabolic data used. However, all these network computational techniques are based on the same assumption that brain regions that function together have correlated activities. Gonzalez-Lima and McIntosh (1994) explicitly stated this assumption as the principle of neural interaction: “It states that if neural regions are synaptically connected, the disturbance in the postsynaptic action potentials of a region is passed on to another. In other words, brain regions do not merely act locally, they interact with one another in complex neural networks. Therefore, brain activity is interdependent on the actions and reactions of the components that form the neural networks.” (Gonzalez-Lima and McIntosh, 1994, p. 24).

Interpreted in terms of neural network analysis, an interregional correlation of CO activity represents the degree to which the neuronal synaptic activity between two regions are related to one another, or how they vary together (covariance). As explained by McIntosh and Gonzalez-Lima (1994), a high interregional correlation between regions A and B means that if region A increases its activity, so too will B, in the case of a positive correlation. The brain is unique among other organs in that it is made of interconnected elements critically dependent on CO activity for synaptic communication, from the local synaptic activity of neurons, to interregional connections among ensembles of neurons across brain regions. Communication among neural elements, whether neurons or ensembles, is along these interconnections, and these network communications represent a large-scale biological underpinning of brain function. In other words, a change in interregional CO activity correlations between neural regions comes about through a change in the synaptic influences of one or more input pathways. Therefore, we identified changes in network interactions after 2VO and MB interventions by examining the interregional correlations of CO activity within regions of the brain. As brain regions became progressively less active after 2VO, their synaptic communications became weaker, resulting in a reduced number of significant interregional correlations. As MB facilitated CO activity in brain regions, so the interregional synaptic communications were stronger, resulting in a higher number of significant interregional correlations.

### Methylene Blue Preserves Cytochrome Oxidase Activity and Prevents Neurodegeneration in Chronic Cerebral Hypoperfusion

The enzymatic activity of CO is responsible for the consumption of oxygen by catalyzing the reaction that reduces oxygen into water in the process of oxidative phosphorylation that generates ATP (Erecinska and Silver, 1989). By preserving CO activity MB can improve oxygen consumption even under hypoxic conditions. For example, Huang et al. (2013) demonstrated that MB has a stronger effect under mild hypoxic conditions by comparing normoxia and hypoxia MB effects *in vivo*. MB under hypoxia induced greater stimulus-evoked fMRI responses and oxygen consumption as compared to normoxia. They concluded “Such enhanced potentiation during hypoxia could be one of the mechanisms that accounts for MB’s neuroprotective effects in metabolically stressed conditions reported in the literature (see review Rojas et al., 2012). For example, the higher the metabolic demand for oxygen consumption, the higher the respiratory chain electron flow produced by MB’s electron cycling action in mitochondria (Rojas et al., 2012). Therefore, MB’s effects during hypoxia may potentiate fMRI responses by further increasing mitochondrial electron transport. We predict that more severe (i.e., 9–12% O2) hypoxia could evoke a larger MB effect.” Furthermore, MB has been shown to protect against brain injury after cardiac arrest, which produces hypoxia-reperfusion injury demonstrated by a decrease in the plasma level of protein S-100Beta, an astroglial marker of hypoxic brain injury (Miclescu et al., 2006).

While it is outside the scope of this paper to provide a comprehensive review of all the properties and effects of MB, we refer the reader to a recent paper that reviews ways in which MB may be neuroprotective by preserving mitochondrial function (Tucker et al., 2018). Similarly, more recently other MB studies have also shown MB’s protective action in multiple models and tissues. For example, Tian et al. (2018) showed that MB protects the lungs from ischemia-reperfusion injury by attenuating mitochondrial oxidative damage. Bhurtel et al. (2018) showed that MB protects dopaminergic neurons against MPTP-induced neurotoxicity by up-regulating brain-derived neurotrophic factor and Biju et al. (2018) showed that MB reduces motor deficits and olfactory dysfunction in a chronic MPTP-probenecid mouse model of Parkinson’s disease. Relevant to our study, Huang et al. (2018) investigated chronic oral MB treatment in a rat model of focal cerebral ischemia-reperfusion. However, the present study is the first to investigate MB neuroprotective effects on mitochondrial CO activity in the pathogenesis of chronic brain hypoperfusion.

When administered during chronic cerebral hypoperfusion, MB preserved mitochondrial CO activity in affected brain regions (secondary motor cortex, perirhinal cortex, primary visual cortex, basolateral amygdala, medial amygdala and hippocampus). This effect was so robust that only one region (secondary visual cortex) that showed decreased CO activity as a result of 2VO was not restored by MB treatment. Additionally, though the same number of subjects showed lesions as a result of 2VO surgery, subjects treated with MB displayed smaller, more localized lesions than subjects treated with saline.

One potential explanation for the appearance of lesions in some subjects and not in others is the induction of necrotic vs. apoptotic mechanisms in response to chronic cerebral hypoperfusion. Necrosis is a form of neuronal cell injury caused by external cellular factors (e.g., toxins or trauma) that result in autolysis, unregulated and detrimental digestion of cellular components. Apoptosis is programmed or targeted cell death, the results of which can potentially benefit the organism. A key determinant of necrosis vs. apoptosis is the presence of intracellular ATP. In the weeks following 2VO surgery, there is a rapid depletion of ATP (Plaschke, 2005; Brīede and Duburs, 2007). This may lead to an induction of necrotic mechanisms in cells more vulnerable to ATP depletion. If this is indeed the case, then increases in ATP production as a result of MB treatment may explain why lesions were smaller and more localized in MB-treated subjects.

Methylene blue treatment in 2VO surgery also resulted in stronger interregional correlation of cytochrome oxidase activity between the same regions that were weakened in saline-treated subjects (i.e., between visual cortex and hippocampus and visual cortex and midbrain). MB treatment in sham and 2VO groups also resulted in widespread strengthened connectivity between hippocampus (CA1, CA2 and dentate) and prefrontal cortex (lateral orbital cortex, ventral orbital cortex and anterior insular cortex), as well as between amygdala (central and medial) and frontal cortex areas (ventral orbital cortex, prelimbic cortex and primary motor cortex). Widespread modulation of interregional correlation of cytochrome oxidase activity by MB has also been reported in the brains of healthy humans analyzed with fMRI (Rodriguez et al., 2017).

Though amygdala-cortical interregional correlation of cytochrome oxidase activity was not significantly affected by 2VO surgery, the strengthened interregional correlation of cytochrome oxidase activity between these regions likely assisted in the learning of the VWT. The amygdala has a modulatory role in both spatial and cued water maze tasks (Packard et al., 1994). The medial amygdala was also positively correlated with VWT performance, strengthening the likelihood that the increased amygdala-cortical interregional correlation of cytochrome oxidase activity we saw as a result of MB treatment was related to learning the VWT escape task.

It has been demonstrated before that low doses (1−4 mg/kg) of methylene blue (1) enhance cytochrome oxidase activity, (2) are neuroprotective, and (3) enhance learning and memory in animals and humans (Rojas et al., 2012; Telch et al., 2014; Echevarria et al., 2016; Rodriguez et al., 2016; Auchter et al., 2017; Zoellner et al., 2017). However, this is the first study to integrate all three of these implications to show that MB is neuroprotective by enhancing mitochondrial activity in regions specifically vulnerable to hypoperfusion. Additionally, functional networks that were specifically weakened by 2VO surgery were restored by daily MB treatment.

## Conclusion

This is the first study to characterize the functional changes in brain metabolic activity that result from chronic cerebral hypoperfusion, and show how treatment with MB ameliorates these changes, preventing neurodegeneration and memory impairment. Interregional correlation of cytochrome oxidase activity was weakened between regions specifically involved in visual discrimination learning, which likely resulted in decreased performance in the visual water task. Treatment with MB not only restored task-specific interregional correlation of cytochrome oxidase activity, but also restored average mitochondrial activity in regions specifically affected by 2VO surgery. These findings implicate MB as a potential prophylactic or therapeutic treatment for chronic cerebral hypoperfusion, including people at a high risk for or symptomatic of neurodegenerative disorders whose pathology is accompanied by cardiovascular disease or cerebrovascular hypoxia.

## Data Availability Statement

The raw data supporting the conclusions of this article will be made available by the authors, without undue reservation, to any qualified researcher.

## Ethics Statement

This study was carried out in accordance with the recommendations of NIH Guide for the Care and Use of Laboratory Animals, Committee for the Update of the Guide for the Care and Use of Laboratory Animals. The protocol was approved by the Institutional Animal Care and Use Committee, University of Texas at Austin.

## Author Contributions

FG-L, AA, and MM designed the experiment and interpreted the results. AA performed the surgery. AA and DB processed the tissue. AA and FG-L analyzed the data. AA, DB, and FG-L wrote the manuscript. FG-L revised and expanded it throughout the peer-review process. All authors contributed to manuscript revision, read and approved the submitted version.

## Conflict of Interest

The authors declare that the research was conducted in the absence of any commercial or financial relationships that could be construed as a potential conflict of interest.
